# Isolation and Identification of the Phenolic Compounds from the Roots of *Sanguisorba officinalis* L. and Their Antioxidant Activities

**DOI:** 10.3390/molecules171213917

**Published:** 2012-11-23

**Authors:** Shuang Zhang, Xin Liu, Zi-Long Zhang, Lu He, Zhe Wang, Guang-Shu Wang

**Affiliations:** School of Pharmaceutical Sciences, Jilin University, Changchun 130021, China

**Keywords:** *Sanguisorba officinalis*, isolation, antioxidant activities, phenolic glycoside, fisetinidol-(4α-8)-catechin, ellagic acid, catechin

## Abstract

Four phenolic compounds were isolated from the roots of *Sanguisorba officinalis* L. by silica gel column chromatography and preparative HPLC. On the basis of chemical and spectroscopic methods, their structures were identified as methyl 4-*O*-β-D-glucopyranosy-5-hydroxy-3-methoxylbenzoate (**1**), 3,3′,4′-tri-*O*-methylellagic acid (**2**), fisetinidol-(4α-8)-catechin (**3**), and (+)-catechin (**4**). Compound **1** is a new phenolic glycoside and compounds **2** and **3** were isolated from the *Sanguisorba* genus for the first time. Compounds **1**–**4** were also assayed for their antioxidant activities using the DPPH free radical assay.

## 1. Introduction

*Sanguisorba officinalis* L. (Rosaceae) is a perennial plant widely distributed in China, and its roots have been used as a traditional Chinese medicine for the treatment of hemostasis and inflammation [1]. Until now, thirty two phenolic compounds, including tannins and flavonoids, were isolated from *S. officinalis* L. [2,3,4,5,6,7]. Pharmacological studies on its hemostatic and anti-inflammatory properties have been reported [8,9], but the molecular level mechanisms of these activities have not been reported until now. In order to study the mechanism of hemostasis, we have carried out the isolation and identification of bioactive constituents of the roots of *S. officinalis* L. In a previous paper [10], we have reported the terpenoid constituents from the roots of *S. officinalis* L. As another part of our study, we report in the present study the isolation and identification of a new phenolic glycoside **1**, together with three phenolic compounds **2**, **3** and **4**, and their antioxidant activity.

## 2. Results and Discussion

### 2.1. Isolation and Identification of Compounds **1–4**

**Figure 1:**
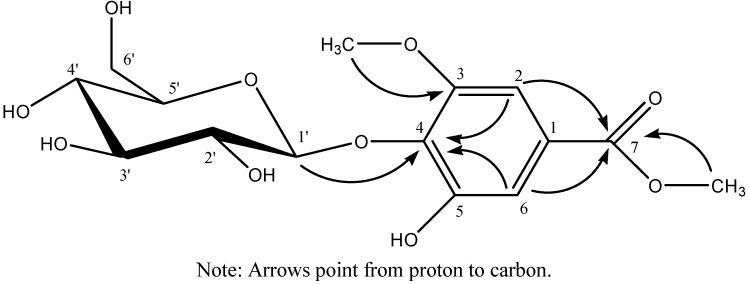
The key HMBC correlations of methyl 4-*O*-β-D-glucopyranosy-5-hydroxy-3-methoxylbenzoate.

Compound **1** was obtained as a colorless amorphous powder, which produced a positive reaction to FeCl_3_ reagent. HR-MS(ESI) indicated the molecular formula of **1** to be C_15_H_20_O_10_. Its IR spectrum indicated the presence of hydroxyl, carbonyl and aromatic groups. Acid hydrolysis of **1** afforded sugar component identified as D-glucose by TLC comparison with an authentic sample. The ^1^H-NMR spectrum (DMSO-*d_6_*) showed the presence of two aromatic protons at δ 7.03 (s, 1H) and 6.92 (s, 1H), two methyl groups at δ 3.77 (s, 3H) and 3.79 (s, 3H), and one β-glucopyranose unit from the anomeric proton at δ 4.83 (d, *J =* 6.0 Hz). The ^13^C-NMR spectrum (DMSO-*d_6_*) showed fifteen carbon signals, among which six are assigned to one sugar unit, nine to the aglycone moiety, and the nine aglycone moiety carbon signals were attributed to two methyl signals, two methine signals and five quaternary carbons by DEPT and HMQC spectra. By analyzing the ^1^H-and ^13^C-NMR data along with the reported data [11], the aglycone moiety was identified as the derivative of gallic acid. The correlations of one methyl protons at δ 3.77 to C-3 at δ 153.0 and another methyl protons at δ 3.79 to C-7 at δ 166.2 in the HMBC spectrum indicated that one methyl group is connected to C-3 through oxygen and another methyl group to C-7 through oxygen. The HMBC correlation H-1' at δ 4.83 to C-4 at δ 138.8 revealed that the linkage position with the glucose unit is at C-4. The complete assignment of the signals of compound **1** was based on DEPT ^13^C-NMR and 2D NMR of H-H COSY, HMQC and HMBC. All the data of ^1^H, ^13^C, and HMBC NMR of compound **1** see Table 1, and key correlations and the structure of compound **1** see Figure 1. Therefore, the structure of compound **1** was elucidated as methyl 4-*O-*β-D-glucopyranosy-5-hydroxy-3- methoxybenzoate.

**Table 1:** ^1^H-NMR (400 MHz), ^13^C-NMR (100 MHz), HMQC and HMBC data of methyl 4-*O-*β*-*D-glucopyranosy-5-hydroxy-3- methoxylbenzoate (DMSO-*d*_6_, δ ppm).

No.	δC	δH	HMBC (H→C)	No.	δC	δH	HMBC (H→C)
**aglycone**				**glc**			
1	125.0			1ʹ	104.5	4.83 (d, 1H, *J* = 6.0 Hz)	138.8
2	102.9	6.92 (s, 1H)	111.8, 138.8, 166.2	2ʹ	74.0	3.27 (m, 1H)	
3	153.0			3ʹ	76.5	3.22 (m, 1H)	
4	138.8			4ʹ	69.6	3.20 (m, 1H)	
5	152.6			5ʹ	77.3	3.13 (m, 1H)	
6	111.8	7.03 (s, 1H)	102.9, 138.8, 166.2	6ʹ	60.7	3.49 (m, 1H), 3.62 (d-like, 1H, *J* = 10.4 Hz)	
7	166.2						
3-OCH3	56.2	3.77	153.0				
7-OCH3	51.9	3.79	166.2				

All assignments based on extensive 1D and 2D NMR experiments (HMQC, HMBC, ^1^H-^1^H COSY).

Using similar methods as described above, compounds **2**–**4** were identified as 3,3′,4′-tri-*O*-methylellagic acid (**2**) [12], fisetinidol-(4α-8)-catechin (**3**) [13], and (+)-catechin (**4**) [14], respectively.

### 2.2. Antioxidant Activity of Compounds **1–4**

Compounds **1**–**4** were next assayed for their antioxidant activity with the DPPH free radical assay, and the results are shown in Table 2. The data proved that fisetinidol-(4α-8)-catechin showed the strongest antioxidant activity.

**Table 2:** The antioxidant assay data of the isolated compounds.

Compound	IC_50_ (ug/mL)
Methyl 4-*O*-β-D-glucopyranosy-5-hydroxy-3-methoxylbenzoate (**1**)	720 ± 7.3
3,3′,4′-tri-*O*-Methylellagic acid (**2**)	820 ± 7.3
Fisetinidol-(4α-8)-catechin (**3**)	12.3 ± 0.2
(+)-Catechin (**4**)	38.2 ± 0.5

Note: All values are averages of at least three runs in Table 2.

## 3. Experimental

### 3.1. General

IR spectra were recorded on a FT-IR 5DX Nicolet/Nicolet Magna IR-560 spectrometer (Thermo Scientific, Osaka, Japan). ^1^H- and ^13^C-NMR spectra were recorded on a Bruker AV-400 spectrometer (Zürich, Switzerland). HR-ESI-MS were recorded on a Bruker microOTOF-Q II mass spectrometer. Prep. HPLC was performed on a Shimadzu LC-10A equipped with a SPD-10A detector and Gemini 5 μm C_18_ 110A column (250 mm × 10.00 mm, 5 μm, flow rate: 3.0 mL/min). The bioactivities were measured on WFZ UV-2100 ultraviolet visible spectrophotometer (Unico Shanghai Instrument Company Limited, Shanghai, China), using the 1,1-diphenyl-2-picrylhydrazyl free radical (DPPH, Sigma-Aldrich, Shanghai, China). The roots of *S. officinalis* L. were collected in Tong-Hua County in Jilin Province, China. They were identified by Prof. Jing-Min Zhang of the School of Pharmaceutical Sciences, Jilin University, Changchun, China.

### 3.2. Extraction and Isolation

The air-dried the roots of *S. officinalis* (4.0 kg) were extracted with 70% EtOH (ca. 20 L, 24 h, room temperature). The EtOH extract was concentrated under reduced pressure, and the viscous concentrate (420 g) was passed through a D101 polyporous resin column eluting successively with H_2_O, 30% EtOH, 70% EtOH, and 95% EtOH, and by vacuum distillation recovery, four fractions were obtained. The 30% ethanol eluate was further chromatographed repeatedly on silica gel columns and then purified by preparative RP-HPLC with CH_3_OH–H_2_O (30:70) to yield the new compound **1** (20 mg). The 95% ethanol eluate was further chromatographed repeatedly on silica gel columns eluted with CHCl_3_–MeOH–EtOAc–H_2_O (3:1:4:2.5, 3:1:7:1.5) to yield compound **2** (200 mg). The 70% EtOH fraction was subjected to silica gel column chromatography eluted with a stepwise gradient mixture of CHCl_3_–MeOH (9:1; 6:1; 3:1), and finally with MeOH alone, and four fractions I–IV were obtained. Fraction IV was further subjected to a silica gel column eluted with CHCl_3_–MeOH–EtOAc–H_2_O (6.5:5:4:1.7), and three fractions (A, B, C) were obtained. Fraction A was applied to a ODS-A (50 μm, 12 nm, YMC, Kyoto, Japan) column eluted with a stepwise gradient mixture of MeOH–H_2_O (2:3; 3:2; 4:1) to yield compound **3** (230 mg). Fraction C was first separated by a ODS-A (50 μm, 12 nm, YMC, Kyoto, Japan) column eluted with a stepwise gradient mixture of MeOH–H_2_O (2:3; 3:2; 4:1), and then purified by preparative HPLC using MeOH–H_2_O (80:20) to yield compound **4** (20 mg).

*Methyl 4-O-β-D-glucopyranosy-5-hydroxy-3-methoxylbenzoate* (**1**): Colorless amorphous powders, produced a positive reaction to FeCl_3_ reagent, m.p. 212–214 °C. HRESIMS, *m/z*: 383.0948 [M+Na]^+^ (calcd for 383.0949). IR (KBr) ν_max_ cm^−1^: 3375 (OH), 1703 (C=O), 1578, 1502, 1403 (aromatic C=C), 1052 (C–O–C). ^1^H and ^13^C-NMR: See Table 1.

*3,3′,4′-Tri-O-methylellagic acid* (**2**): Pale yellow amorphous powder, produced a positive reaction to FeCl_3_ reagent. ESIMS, *m/z*: 345 [M+H]^+^. ^1^H-NMR (DMSO-*d_6_*) δ: 10.46 (1H, s, C4-OH), 7.53 (1H, s, 5-H), 7.60 (1H, s, 5′-H), 4.07 (3H, s, C3-OCH_3_), 4.09 (3H, s, C3′-OCH_3_), 4.01 (3H, s, C4′-OCH_3_). ^13^C-NMR (DMSO-*d_6_*) δ: 111.6 (C-1), 141.0 (C-2 ), 140.2 (C-3), 152.6 (C-4), 111.2 (C-5), 112.5 (C-6), 158.3 (C-7), 111.9 (C-1′), 141.5 (C-2′), 140.8(C-3′), 153.8 (C-4′), 107.5 (C-5′), 113.4 (C-6′), 158.5 (C-7′); 61.0 (C3-OCH_3_), 61.3 (C3′-OCH_3_), 56.7 (C4′-OCH_3_).

*Fisetinidol-(4α-8)-catechin* (**3**): yellow amorphous powder produced a positive reaction to FeCl_3_ reagent. HRESIMS, *m/z*: 563.1536 [M+H]^+^; (calcd for C_30_H_27_O_11_, 563.1548). ^1^H-NMR (DMSO-*d_6_*) δ: 2.41 (dd, *J =* 16.0 and 9.2 Hz, H-4F), 2.84 (dd, *J =* 16.0 and 5.2 Hz, H-4F), 3.76 (m, H-3F), 4.32 (d, *J =* 8.5 Hz, H-4C), 4.35 (d, *J* = 8.5 Hz, H-2C), 4.45 (t, *J =* 8.5 Hz, H-3C), 4.48 (d, *J =* 8.4 Hz, H-2F), 6.05 (dd, *J =* 8.0 and 1.5 Hz, H-6′E), 5.92(s, H-6D), 6.06 (d, *J =* 2.4 Hz, H-8A), 6.17 (dd, *J =* 8.0 and 2.4 Hz, H-6A), 6.48 (d, *J =* 8.0 Hz, H-5A), 6.65 (dd, *J* = 8.0 and 2.0 Hz, H-6′B and H-6′E), 6.66 (d, *J* = 8.0 Hz, H-5′E and H-5A), 6.68 (d, *J =* 8.0 Hz, H-5′B), 6.76 (d, *J =* 2.0 Hz, H-2′B), 6.80 (d, *J =* 2.0 Hz, H-2′E). ^13^C-NMR (DMSO-*d_6_*) δ: 28.8 (C-4F), 40.0 (C-4C), 66.9 (C-5F), 67.9 (C-3C), 81.4 (C-2F), 82.9 (C-2C), 95.9 (C-6D), 98.6 (C-4aF), 101.9 (C-8A), 106.5 (C-8D), 108.0 (C-6A), 114.7 (C-2′E), 114.8 (C-5′E), 115.0 (C-2′B), 115.2 (C-5′B),118.2 (C-4aC), 118.4 (C-6′E), 119.6 (C-6′B), 128.5 (C-5A), 130.9 (C-1′E), 131.2 (C-1′B), 144.8 (C-3′B and C-3′E), 144.9 (C-4′B and C-4′E), 153.8 (C-7D), 154.2 (C-8aA), 154.7 (C-8aD), 155.0 (C-5D), 156.6 (C-7A).

*(+)-Catechin* (**4**): Pale yellow amorphous powder, produced a positive reaction to FeCl_3_ reagent. ESIMS, *m/z*: 291 [M+H]^+^. ^1^H-NMR (DMSO-*d_6_*) δ: 2.35 (1H, dd, *J =* 15.9, 7.8 Hz, H-4), 2.66 (1H, dd, *J =* 15.9, 4.8 Hz, H-4), 3.81 (1H, m, H-3), 4.48 (1H, d, *J =* 7.3 Hz, H-2), 5.69 (1H, s, H-8), 5.89 (1H, s, H-6), 6.59 (1H, d, *J =* 7.8 Hz, H-6′), 6.68 (1H, d, *J =* 7.8 Hz, H-5′), 6.72 (1H, s, H-2′). ^13^C-NMR (DMSO-*d_6_*) δ: 27.8 (C-4), 66.3 (C-3), 81.0 (C-1), 93.8 (C-8), 95.1 (C-6), 99.0 (C-4a), 114.5 (C-2′), 115.0 (C-5′), 118.4 (C-6′), 130.6 (C-1′), 144.8 (C-3′,4′), 155.3 (C-8a), 156.1 (C-5), 156.4 (C-7).

### 3.3. Acid Hydrolysis of **1**

Solution of **1**(1.0 mg) in 0.5 M H_2_SO_4_ (2.0 mL) was heated under reflux for 3 h. After cooling, the reaction mixture was diluted with H_2_O, neutralized with BaCO_3_, then filtered. The solution was partitioned with EtOAc to give two layers. The aqueous layer was evaporated and then subjected to TLC analysis with authentic sugar samples using *n*-BuOH–MeOH–CHCl_3_–HOAc (12.5:4.5:9:1.5:1, detection with aniline-phthalic acid). Compounds **1** afforded D-glucose (R_f_ = 0.30).

### 3.4. Bioactivity Assay

The antioxidant activity of compounds **1**–**4** were assessed according to their DPPH scavenging ability. Reaction mixtures, containing 0.5 mL of the relevant compound (dissolved in EtOH) and 2.5 mL of a 100 µM DPPH ethanolic solution, were added to 96-well microtiter plates and incubated at 37 °C for 30 min. Absorbances were measured at 515 nm. Percent inhibition was determined by comparison with an EtOH-treated control group. IC_50_ values denote the concentration of samples required to scavenge 50% of the DPPH free radicals.

## 4. Conclusions

Compound **1** is a new phenolic glycoside and compounds **2** and **3** were isolated from the *Sanguisorba* genus for the first time. Compounds **1**–**4** were assayed for their antioxidant activity with DPPH free radicals, and the data proved that fisetinidol-(4α-8)-catechin showed the strongest antioxidant activity. 
